# Detection of Congestive Heart Failure Based on LSTM-Based Deep Network via Short-Term RR Intervals

**DOI:** 10.3390/s19071502

**Published:** 2019-03-28

**Authors:** Ludi Wang, Xiaoguang Zhou

**Affiliations:** Automation School, Beijing University of Posts and Telecommunications, No. 10 Xitucheng Road, Beijing 100876, China; zxg@bupt.edu.cn

**Keywords:** short-term RR intervals, congestive heart failure, deep learning, inception module

## Abstract

Congestive heart failure (CHF) refers to the inadequate blood filling function of the ventricular pump and it may cause an insufficient heart discharge volume that fails to meet the needs of body metabolism. Heart rate variability (HRV) based on the RR interval is a proven effective predictor of CHF. Short-term HRV has been used widely in many healthcare applications to monitor patients’ health, especially in combination with mobile phones and smart watches. Inspired by the inception module from GoogLeNet, we combined long short-term memory (LSTM) and an Inception module for CHF detection. Five open-source databases were used for training and testing, and three RR segment length types (N = 500, 1000 and 2000) were used for the comparison with other studies. With blindfold validation, the proposed method achieved 99.22%, 98.85% and 98.92% accuracy using the Beth Israel Deaconess Medical Center (BIDMC) CHF, normal sinus rhythm (NSR) and the Fantasia database (FD) databases and 82.51%, 86.68% and 87.55% accuracy using the NSR-RR and CHF-RR databases, with N = 500, 1000 and 2000 length RR interval segments, respectively. Our end-to-end system can help clinicians to detect CHF using short-term assessment of the heartbeat. It can be installed in healthcare applications to monitor the status of human heart.

## 1. Introduction

Heart failure (HF) is a clinical syndrome of various heart diseases at severe stages and is also known as congestive heart failure (CHF). It is caused by an inadequate blood filling function of the ventricular pump. A poor heart pump function can cause the heart’s discharge volume to be insufficient to meet the needs of the body’s metabolism. Additionally, the blood perfusion of tissues and organs becomes insufficient, and there may be congestion of pulmonary and general circulation. Heart failure is categorized into four levels by the New York Heart Association (NYHA). Only patients at levels III and IV have significant symptoms [1]. Worldwide, more than 23 million patients are affected by heart failure, which makes it a major public health problem and huge economic burden [2]. In the USA, the total cost of nursing HF patients is $ 31 billion and this figure is estimated to increase to $70 billion by 2030 [3]. In addition, the treatment of heart disease comprises the highest health care costs of low- and middle-income countries.

Echocardiography is often used to diagnose CHF in hospitals. This instrument uses ultrasound to measure the stroke volume, end diastolic volume and the ratio between these two quantities, which is also known as the ejection fraction. The general ejection fraction should be between 50% and 70%, and is less than 40% in the chronic systolic HF. The other method for detecting CHF is by using an electrocardiogram (ECG). The standard 12-lead ECG remains the most useful instrument in the diagnosis and prognosis of CHF patients. However, the reliability of diagnoses could be further enhanced by signal processing techniques and biomedical analysis [4]. In recent years, many researchers have worked on CHF detection using ECG. For instance, Dhingra et al. [5] showed that longer electrocardiographic QRS duration is associated with CHF. Larisa et al. [6] used beat-to-beat QT variability to separate the healthy individuals from HF subjects. Among the recent methods, HRV analysis has attracted wide attention for its potential to detect CHF. Nolan et al. reported the standard deviation of the heart rate (SDNN) can effectively predict the risk of mortality for CHF [7]. Binkey et al. detected parasympathetic withdrawal by noninvasive HRV spectral analysis and found that this feature is a part of the autonomic nervous disorder feature in CHF patients [8]. Yu and Lee detected CHF with bispectral analysis and a genetic algorithm (GA) [9]. Woo et al. found that Poincare plot analysis can detect labeled sympathetic nerve activation in CHF patients and found a link between autonomic nerve change and sudden cardiac death [10]. Peng et al. demonstrated a reduction in HR complexity in CHF subjects based on fractal dimension analysis (FDA) [11]. Chen et al. proposed a dynamic HRV to describe the dynamic fluctuation of HRV over a 24-h period, and achieved over 95% accuracy [12]. However, they admitted that the robustness of HRV-based approaches remains an issue to be addressed since its sensitivity changes according to the clinical condition [13].

However, most of the above studies required long-term RR interval data, which is not possible in health-care situations outside of hospital, especially with the heart rate testing applications currently being developed for use with mobile devices like smartphones. The short-term HRV analysis may be useful in monitoring dynamic changes to cardiac autonomic activity [14]. It has been used to detect cardiovascular diseases such as atrial fibrillation (AF) and achieved good results [15]. Oliver Faust et al. [16] used a long short-term memory network with RR intervals as signals for the automated detection of atrial fibrillation, and received 99.77% accuracy with blindfold validation. Thakre and Smith [17] observed that the lag-response of Poincaré plot indices are related to CHF. Liu et al. presented an entropy (FuzzyMEn) method to classify normal and CHF patients [18], and a comparison of the accuracy of entropy arguments on CHF subjects can be found in [19]. Liu et al. [20] also tried to detect CHF with short-term RR intervals using multiscale entropy analysis based on RR interval signals and a support vector machine (SVM) classifier. Yoon et al. [21] achieved 84.49% accuracy in CHF detection by observing just 16 heartbeats. They extracted three “expert features”—normalized root mean squared successive difference (RMSSD), sample entropy and Shannon entropy—and used threshold values of these to detect CHF.

Besides, they concentrated on subjects with severe CHF (NYHA class III-IV) since the RR intervals they used was from the Beth Israel Deaconess Medical Center (BIDMC) congestive heart failure database [22,23]. However, the detection of the CHF patients with I-II class is equally significant, especially for daily care.

Deep learning [24,25] has been applied in various fields, such as image recognition and speech recognition, and achieved remarkable results [25,26,27,28]. In recent years, certain scholars have applied deep learning to the recognition of ECG signals. Chen et al. provided a CHF detection method based on sparse auto-encoder deep learning (SAE-based DL) of short-term RR intervals [13]. Potes et al. [29] used an ensemble of feature-based and deep learning-based classifiers for detection of abnormal heart sounds. Hwang et al. [30] provided an optimal deep learning framework for monitoring mental stress using ultra-short-term ECG signals. Pourbabaee et al. [31] used deep convolution neural networks to learn ECG features for screening paroxysmal atrial fibrillation patients. Since the decision-making system based on deep learning obtains all the information with the data, there is no information reduction through feature extraction.

Inspired by the Inception module introduced by GoogLeNet [32], we combined the long short-term memory (LSTM) network [27] and convolution net architecture [33] to construct the diagnosis system network structure and detect CHF automatically. As a result, we proposed an end-to-end system based on deep learning for CHF detection via short-term RR interval, using five open-source databases containing all-class CHF data.

The paper is organized as follows. Section 2 presents a detailed description of the proposed method, including the database used, network topology, the basic steps and evaluation methods. The classification results are presented in Section 3. Section 4 provides a discussion and describes the limitations of the study, and Section 5 presents the conclusion.

## 2. Materials and Methods

### 2.1. Data

In this study, five open-source databases were used to evaluate the proposed method. For CHF patients, the BIDMC congestive heart failure database (BIDMC-CHF) [22,23] and congestive heart failure RR interval database (CHF-RR) [22], available on PhysioBank, were used. The BIDMC-CHF dataset has 15 subjects (11 men, aged 22–71 years, and four women, aged 54–63) with severe CHF (NYHA class 3–4), and the CHF-RRI dataset includes 29 recordings of subjects aged 34–79 with CHF (NYHA class 1–3). For normal subjects, the Massachusetts Institute of Technology-Beth Israel Hospital (MIT-BIH) normal sinus rhythm (NSR) [22], the Fantasia database (FD) [34] and the normal sinus rhythm RR interval database (NSR-RR) [22] were used. The NSR includes 18 subjects with no significant arrhythmias (5 men, aged 26–45, and 13 women, aged 20–50), the NSR2 includes beat annotation files for 54 subjects with normal sinus rhythm (30 men, aged 28.5–76, and 24 women, aged 58–73) and the FD includes 120min continuous ECG signals of 20 young and 20 elderly healthy subjects.

**Figure 1:**
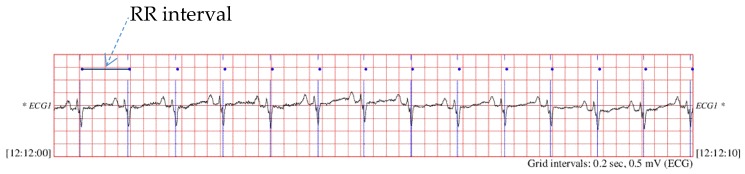
The original ECG signals and beat annotations.

**Figure 2:**
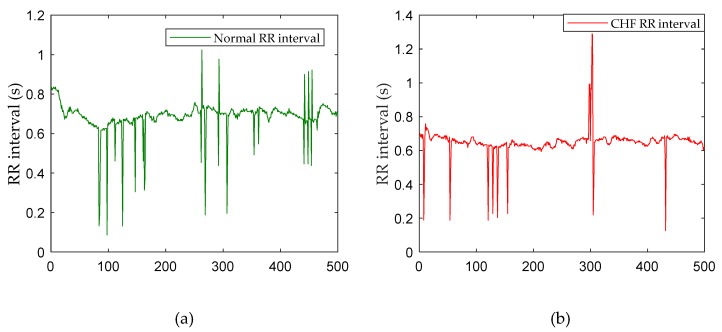
Signals of different type for 500 samples length. (**a**) The normal RR interval. (**b**) The congestive heart failure (CHF)-RR interval.

As shown in Figure 1, the original signals in above databases are ECG recordings. These databases also included beat annotation obtained by automated analysis with manual review and correction. In this study, we used those beat annotations to extract the RR interval (the time interval between two adjacent R-wave amplitudes) as the input signals. In order to compare our results with other work, we segmented the data into 500, 1000 and 2000 beats, which means the input signal in this model was a sequence of 500, 1000 and 2000 time values in seconds. Table 1 summarizes the number of signals for the different databases in two classes. Figure 2 shows the signals of different types for the 500 sample length (measured in seconds).

### 2.2. LSTM-Based Deep Convolutional Neural Network Structure

Inception was first introduced by the GoogLeNet [32]. Its main advantage is that it can get significant quality gain in the moderate increase of computing demand, compared with the lighter and wider network. The name of Inception” was derived from the network in the network paper by Lin et al. [35]. The Inception will calculate the different transformations from one input at the same time and output the results to the next level. As a result, the model itself can decide whether to use the information or what information to use. The first version of Inception was GoogLeNet, also known as the 22 layers network that won the ILSVRC 2014 competition. A year later, the researchers developed Inception V2 and V3 in second papers, and achieved a variety of improvements in the original version—the most important thing to mention was that they made the larger convolution weight a continuous and smaller convolution, making learning easier.

**Figure 3:**
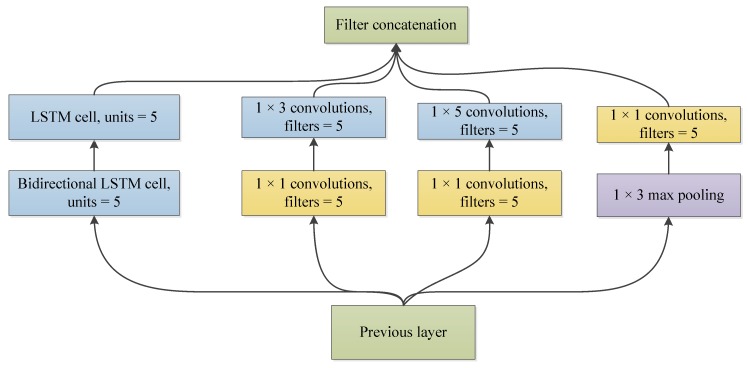
Inception—long short-term memory (LSTM) module used in this paper.

**Figure 4:**
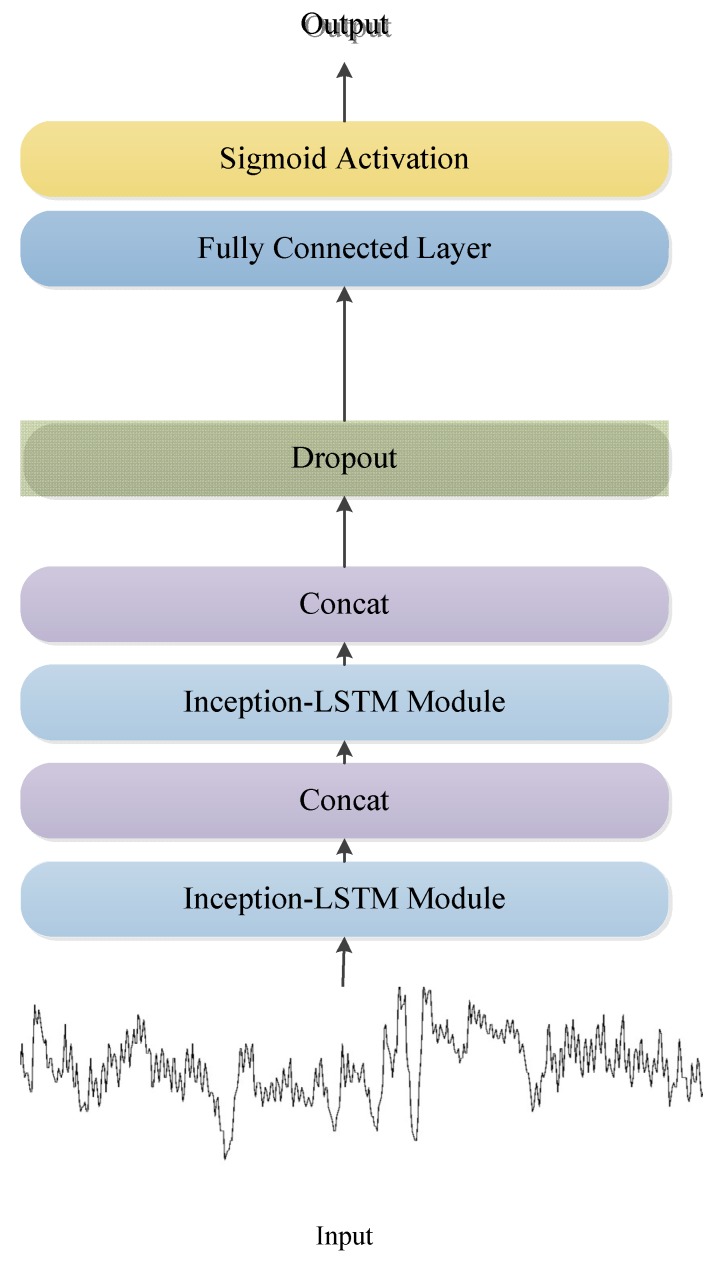
Network structure in this method, the input is RR intervals.

**Figure 5:**
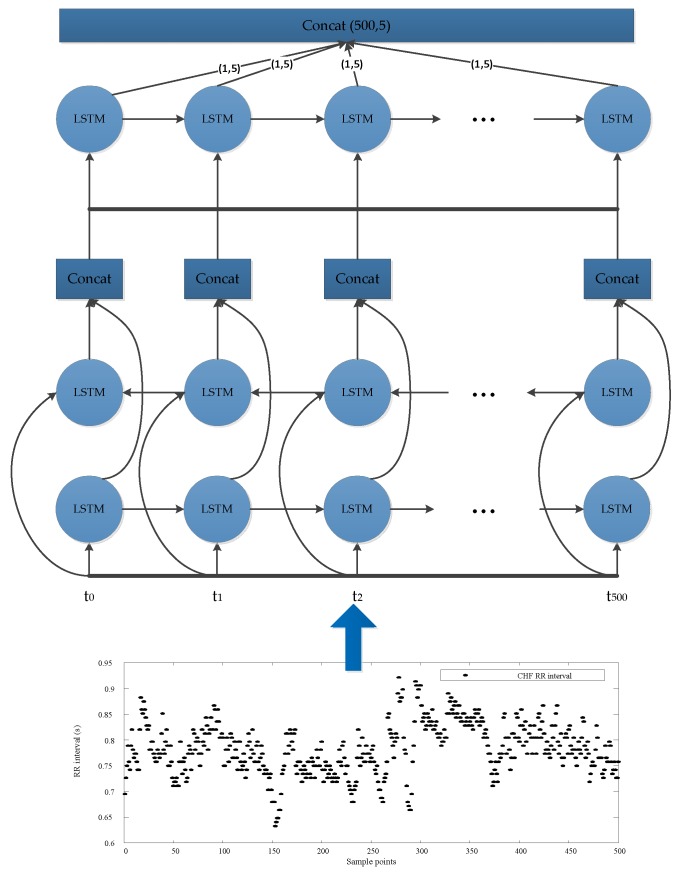
The LSTM network used in the module (with many-to-many structure, the input data is 500 RR interval segments).

**Figure 6:**
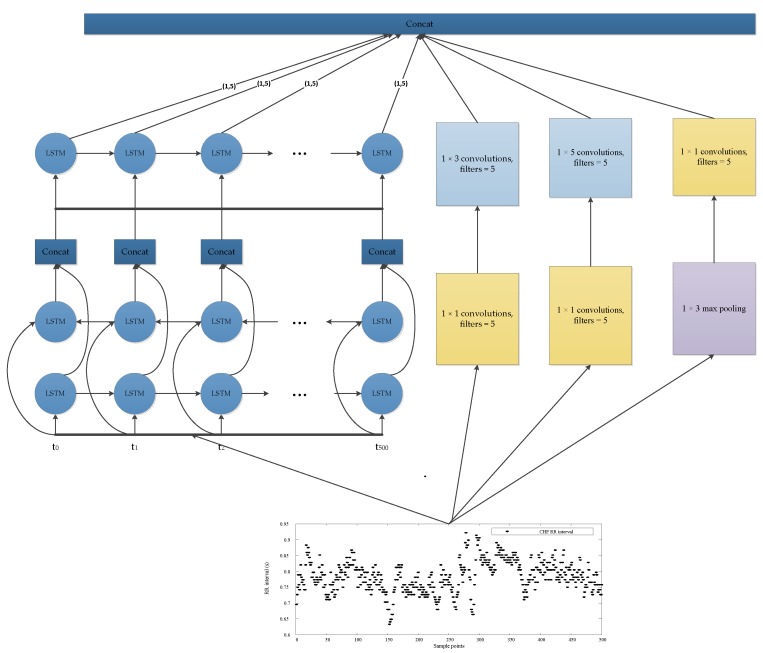
The detailed structure of the module in this model (the input data is 500 RR interval segments).

**Figure 7:**
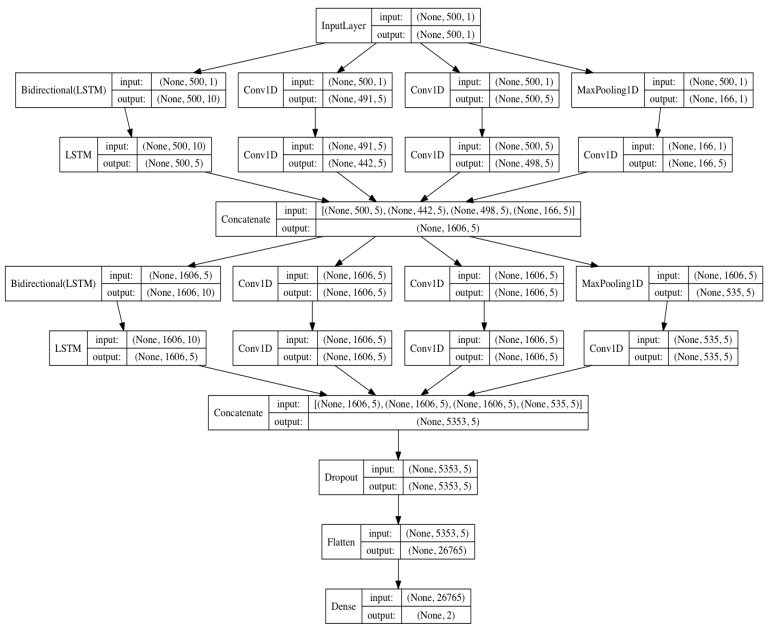
The details of the proposed network structure with 500 RR intervals.

In this study, we used an LSTM network [36] to replace one of the convolution networks in the Inception module, as shown in Figure 3. LSTM modules have received great results in the detection of time series signals, including RR interval signals [16]. Since the low complexity of the heart rate signal, only two Inception–LSTM modules were used, as shown in Figure 4 and Table 2. We used the LSTM with many-to-many structures as a feature extractor, as shown in Figure 5. Figure 6 presents the detailed structure of one Inception–LSTM module we used, and Figure 7 is the detailed network structure of the proposed model with 500 length RR intervals. For preventing overfitting, a dropout layer was used in this paper, and we set the rate = 0.2. We also used Adam (short for adaptive moment estimation) as the optimizer. In this optimization algorithm, running averages of both gradients and the second moments of the gradients were used. We set the parameters the same as the paper [37], namely: learning rate = 0.001, β1 = 0.9 and β2 = 0.999. In this study, we took the CHF patients as positive subjects and NSR persons as negative subjects, and then classified the input data into these two categories by sigmoid activation function. Since the sigmoid function was used as the activation in output layer, we used binary cross entropy as the loss function:

*L*(*y*,*p*) = −*logPr*(*y*|*p*) = −(*ylog*(*p*) + (1 − *y*)*log*(1 − *p*)), (1)

where *y* is the true label and *p* is the prediction.

### 2.3. Evaluation Method

In this study, three indicators were used for testing: sensitivity, specificity and accuracy. The definitions of above three indices are as follows:

*Sensitivity* = *TP*/(*TP* + *FN*) *Specificity* = *TN*/(*TN* + *FP*) *Accuracy* = (*TP* + *TN*)/(*TP* + *FP* + *TN* + *FN*), (2)

where *TP* is the number of true positives, *FN* is the number of false negatives, *FP* is the number of false positives, and *TN* is the number of true negatives.

## 3. Results

To better verify the proposed method, we compared the results of the proposed approach with those of other studies. However, other studies used different datasets to verify their methods. Liu et al. [20] used the normal sinus rhythm RR interval database (NSR-RR) and congestive heart failure RR interval database (CHF-RR), while Chen et al. [13] used the 5-min RR interval. Kumar [38] used the BIDMC-CHF database, MIT-BIH NSR database and Fantasia dataset for CHF detection. Therefore, in this study, for examining the proposed method, we used the same datasets for comparison, which as shown in Table 3.

It can be seen from the previous studies that the classification performance using database 1 (DB1) is better than the performance using database 2 (DB2). The main reason may be the subjects in the DB1 (NYHA classes 3–4) suffered more severe CHF than the subjects in the DB2 (NYHA classes 1–3). Therefore, the variability of the signal in the DB1 is more obvious and easier to be detected.

### 3.1. 10-Fold Cross-Validation Stage

**Figure 8:**
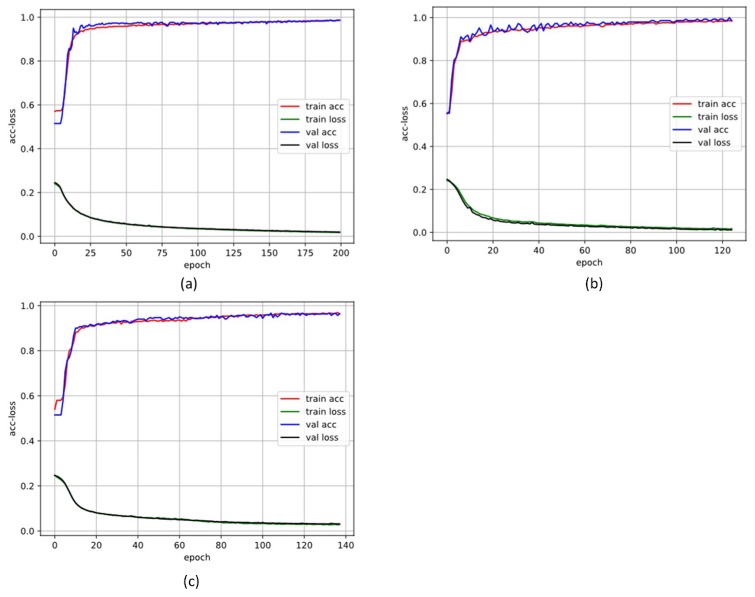
Training and validation loss function over the epochs in database 1 (DB1). (**a**) The 500 length segment; (**b**) the 1000 length segment; (**c**) the 2000 length segment.

**Figure 9:**
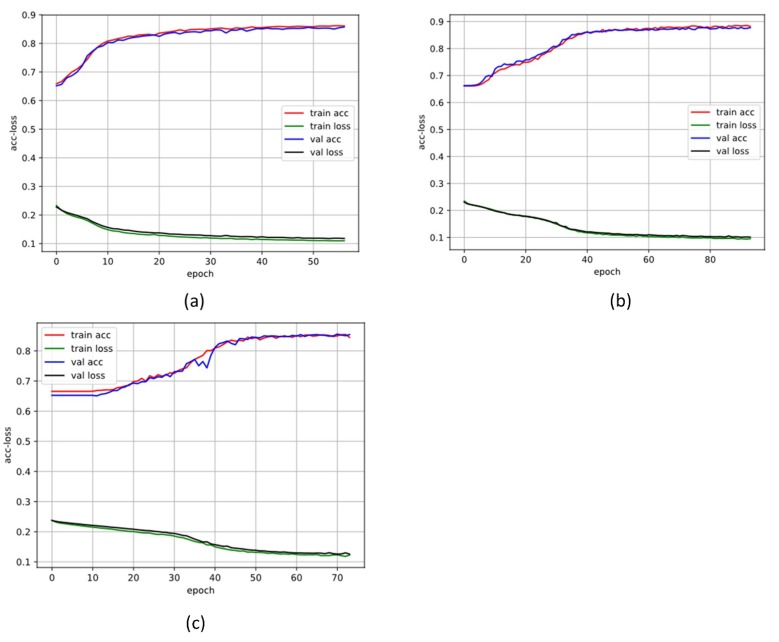
Training and validation loss function over the epochs (DB2). (**a**) The 500 length segment; (**b**) the 1000 length segment; (**c**) the 2000 length segment.

In the training stage, 10-cross validation and early stopping method were used for preventing overfitting. We first shuffled all the signals, and then split them as training segments and validation segments. The training segments were randomly shuffled again at each epoch (the validation segments were not). The early stopping method stops training when the validation loss has stopped improving. Figure 8 and Figure 9 show the training process details of different training datasets; the solid line is the mean of the performance for each of 10-folds. We also set the batch size as 128 in the training. The batch means a set of N (128) samples, and a batch results in only one update to the model. The max epochs were set as 100. In the training stage for each fold, the training details and parameters are as listed in Table 4.

For comparison, we also used three other methods from the reference [13,20,38]. It is worthy to note that the mentioned methods [13,20,38] used cross-validation for testing instead of a blind testing method. As a result, we compared the results in this stage. In addition, we also used the same model without LSTM units for evaluating the effect of introducing it to the original inception. The overall performance of the training process and comparison are listed in Table 5 and Table 6.

### 3.2. Blind Fold Testing Results

The common way to model validation is by k-fold cross validation or split validation. However, in those literatures, the input signals were independent of each other. For example, there was only one photo of subject A, and it can only appear in the training dataset or the testing dataset. In this study and comparison studies mentioned above, the original RR intervals were segmented by different length (500, 1000 and 2000). It means that there were multiple RR interval segments of one subject, and they can both appear in training dataset and testing dataset if we use split validation, although these two RR interval segments were not exactly the same.

In practice, the classification system had to deal with completely unknown subjects and not with unknown signal sequences of otherwise known subjects, as in the case of cross validation or split validation. Therefore, we used blindfold testing to better evaluate the proposed method. The blindfold dataset consists of the RR intervals from the subjects who never appeared in the training stage, and thus reduce the possibility of over-fitting. To the best of our knowledge, we were the first to use this method in testing stage for detecting CHF. Blindfold testing can effectively verify the performance of the proposed classification system when dealing with completely unknown subjects. The information of the subjects in the blindfold testing dataset are listed in Table 7. The results and comparisons are provided in Table 8 for different dataset.

From results, it is observed that the model with the modified Inception performed better than the comparison method. One reason is that LSTM units improve the handling of time step information from input sequences by incorporating a gating mechanism. Because of there can be lags of unknown duration between important events in a time series, LSTM networks are well-suite for the classification and process of time series signal [16].

## 4. Discussion

Inspired by GoogLeNet [32], a deep learning network using an LSTM-based Inception module for CHF detection, via short-term RR interval was proposed in this study. Five open-source databases and three types of RR segment length (N = 500, 1000 and 2000) were used to better evaluate the proposed method and compare with other studies. With blindfold validation, the proposed method achieved 99.22%, 98.85% and 98.92% accuracy on N = 500, 1000 and 2000 length RR intervals, respectively, using the BIDMC-CHF, NSR and FD databases; and achieved 82.51%, 86.68% and 87.55% accuracy on N = 500, 1000 and 2000 length RR intervals, respectively, using the NSR-RR and CHF-RR databases.

A possible explanation for the better performance of our method is that the deep-learning features allow more reliable signal abstraction in high dimensional space without human operation. The deep learning system forms a more abstract high-level representation of attribute classes or features by combing low-level features to discover distributed feature representations of data.

The proposed system can be installed inlow-cost ECG devices and be a diagnostic tool in places where access to a cardiologist is difficult. This system can also send the preliminary diagnostic results to cardiologists via the internet to save expert clinicians and cardiologists considerable time and decrease the number of misdiagnoses.

There two advantages of the present method. Firstly, deep learning method was used to CHF detection. Since the decision-making system based on deep learning gets all the information with the data, there is no information reduction through feature extraction. Therefore, our method can avoid potential error and automatically diagnose CHF. Secondly, we modified the inception module by the LSTM, which is well-suited to classifying time series signal, since there can be lags of unknown duration between import events in such data.

However, there are several limitations to this study. First, we did not focus on the problem of data imbalance. Table 1 shows that the sample sizes of healthy and CHF subjects are uneven, especially for the experiments using the CHF-RR and NSR-RR datasets. Secondly, the present method required big data and more computational power to train the model and obtain the optimum performance.

## 5. Conclusions

In summary, this study proposed an automated classifier for CHF detection that achieved good classification performance. The blindfold testing method was used to better evaluate the performance of the method in the situation of dealing with completely unknown subjects, which is more in line with reality. Using short-term HRV signals to detect CHF is important for healthcare applications, especially for smartphones and smart watches. This method can help clinicians monitor CHF patients outside the hospital and better make sense of HRV signals. We also hope this study can provide technical support for the identification and management of CHF patients based mobile phones.

In our future work, we will try to solve the data imbalance issue and other deep learning method for CHF detection, such as attention network. In addition, we will apply the model to smart watch or mobile application, and use it as a routine clinical application to assist doctors. The model will first give a preliminary diagnosis to users, then receive the doctor’s review and correction, and re-train the model based on the new input data. We expect the method to be a useful automatic tool to increase the detection rate of patients with CHF.

## Figures and Tables

**Table 1:** The number of signals for different database in two classes.

Database	Total Segments		
	N = 500	N = 1000	N = 2000
BIDMC congestive heart failure database (CHF)	3214	1607	803
Congestive heart failure RR interval database (CHF)	6622	3311	1655
MIT-BIH normal sinus rhythm database (NSR)	3579	1739	869
Normal sinus rhythm RR interval database (NSR)	11,583	5791	2895
Fantasia dataset (NSR)	500	250	125

**Table 2:** The detailed structure of the proposed model.

Layer	Type	Depth	Segment Length	Output Shape
0	Input layer	0	500	500 × 1
			1000	1000 × 1
			2000	2000 × 1
0–1	Inception-LSTM module#1	2	500	1606 × 5
			1000	3327 × 5
			2000	6660 × 5
1–2	Concatenate layer			
2–3	Inception-LSTM module#2	2	500	5353 × 5
			1000	11,090 × 5
			2000	22,200 × 5
3–4	Concatenate layer			
4–5	Dropout	0	-	
5–6	fully connected	1	500	26,765
			1000	55,450
			2000	111,000
6	Sigmoid	0		2

**Table 3:** Dataset used for comparison.

Database	BIDMC-CHF	CHF-RR	MIT-BIH NSR	NSR-RR	Fantasia
Database-1 (DB1)	√		√		√
Database-2 (DB2)		√		√	

**Table 4:** Training details and parameters.

Parameters	Value
Shuffled	True
Batch size	128
Max epochs	100
Early stopping	monitor = validation loss, patience = 5
Loss function	Binary entropy
Optimizer	Adaptive moment estimation

**Table 5:** Performance of the 10-fold cross-validation (DB1).

Method	Classifier	Features	Length	Evaluation		
				Sensitivity	Specificity	Accuracy
[38]	LS-SVM	Accumulated fuzzy entropy and accumulated permutation entropy	500	98.07%	98.33%	98.21%
			1000	97.95%	98.07%	98.01%
			2000	97.76%	97.67%	97.71%
This paper	Inception module	-	500	97.80%	98.16%	97.96%
			1000	98.67%	96.69%	97.84%
			2000	93.82%	100.00%	96.75%
	LSTM based Inception	-	500	99.45%	98.91%	99.14%
			1000	97.74%	98.72%	98.31%
			2000	97.64%	99.83%	98.69%

**Table 6:** Performance of the 10-fold cross-validation (DB2).

Method	Classifier	Features	Length	Evaluation		
				Sensitivity	Specificity	Accuracy
[13]	DNNs	Sparse-auto-encoder	500	49.09%	86.33%	72.86%
[20]	SVM	Multiscale entropy of ΔRR	1000	86.2%	85.2%	85.5%
			2000	84.4%	86.8%	85.6%
This paper	Inception module	-	500	97.38%	30.14%	74.32%
			1000	86.38%	58.31%	76.56%
			2000	87.87%	62.93%	79.31%
	LSTM based Inception	-	500	91.21%	74.91%	86.42%
			1000	92.07%	76.47%	87.76%
			2000	90.83%	77.65%	86.63%

**Table 7:** Information of the blindfold testing dataset.

Database	Blind Validation Dataset				
	Subject Information (Age, Sex, Number)		Total Segments		
	CHF	Normal	N = 500	N = 1000	N = 2000
Database-1 (DB1)	(54, F, #11) (63, M, #13) (61, M, #14)	(50, F, #19830) (38, F, #19140) (34, M, #19093)	686	339	164
Database-2 (DB2)	(35, unknown, #224) (66, unknown, #225) (51, unknown, #226) (64, unknown, #227) (51, unknown, #228) (58, unknown, #229)	(39, M, #049) (29, M, #050) (40, M, #051) (35, M, #054) (64, F, #001) (67, F, #003)	2707	1343	662

**Table 8:** Results of blindfold testing.

Dataset	Segment Length	Evaluation		
		Sensitivity	Specificity	Accuracy
DB1	500	99.22%	99.72%	99.22%
	1000	98.13%	100.00%	98.85%
	2000	98.85%	98.99%	98.92%
DB2	500	91.90%	73.58%	82.51%
	1000	96.85%	75.82%	86.68%
	2000	94.14%	81.25%	87.55%
